# An Investigation into the Relationship of Circulating Gut Microbiome Molecules and Inflammatory Markers with the Risk of Incident Dementia in Later Life

**DOI:** 10.1007/s12035-023-03513-6

**Published:** 2023-08-22

**Authors:** Kolade Oluwagbemigun, Andrea Anesi, Urska Vrhovsek, Fulvio Mattivi, Pamela Martino Adami, Michael Pentzek, Martin Scherer, Steffi G. Riedel-Heller, Siegfried Weyerer, Horst Bickel, Birgitt Wiese, Matthias Schmid, John F. Cryan, Alfredo Ramirez, Michael Wagner, Ute Nöthlings

**Affiliations:** 1 Nutritional Epidemiology, Department of Nutrition and Food Sciences, University of Bonn, 53115 Bonn, Germany; 2 Department of Food Quality and Nutrition, Research and Innovation Centre, Fondazione Edmund Mach (FEM), 38098 San Michele all’Adige, Italy; 3 Division of Neurogenetics and Molecular Psychiatry, Department of Psychiatry and Psychotherapy, Medical Faculty, University of Cologne, 50924 Cologne, Germany; 4 Institute of General Practice, University Hospital Essen, 45147 Essen, Germany; 5 Department of Primary Medical Care, Center for Psychosocial Medicine, University Medical Center, 20246 Hamburg-Eppendorf, Germany; 6 Institute of Social Medicine, Occupational Health and Public Health, University of Leipzig, 04103 Leipzig, Germany; 7 Medical Faculty Mannheim, Heidelberg University, 68167 Mannheim, Germany; 8 Department of Psychiatry, Technical University of Munich, 80336 Munich, Germany; 9 Institute of General Practice, Hannover Medical School, 30625 Hannover, Germany; 10 Institute for Medical Biometry, Informatics and Epidemiology, Faculty of Medicine, University of Bonn, 53127 Bonn, Germany; 11 German Center for Neurodegenerative Diseases, 53127 Bonn, Germany; 12 Department of Anatomy and Neuroscience, Western Gateway Building, University College Cork, Cork, T12 XF62 Ireland; 13 Department of Neurodegenerative Diseases and Geriatric Psychiatry, University Hospital Bonn, 53127 Bonn, Germany; 14 Department of Psychiatry and Glenn Biggs Institute for Alzheimer’s and Neurodegenerative Diseases, San Antonio, TX 78229 USA; 15 Excellence Cluster on Cellular Stress Responses in Aging-Associated Diseases (CECAD), University of Cologne, 50931 Cologne, Germany

**Keywords:** Gut microbiome molecules, Lipopolysaccharide, Short-chain fatty acids, Indole-containing tryptophan metabolites, Systemic inflammatory markers, Dementia

## Abstract

The gut microbiome may be involved in the occurrence of dementia primarily through the molecular mechanisms of producing bioactive molecules and promoting inflammation. Epidemiological evidence linking gut microbiome molecules and inflammatory markers to dementia risk has been mixed, and the intricate interplay between these groups of biomarkers suggests that their joint investigation in the context of dementia is warranted. We aimed to simultaneously investigate the association of circulating levels of selected gut microbiome molecules and inflammatory markers with dementia risk. This case–cohort epidemiological study included 805 individuals (83 years, 66% women) free of dementia at baseline. Plasma levels of 19 selected gut microbiome molecules comprising lipopolysaccharide, short-chain fatty acids, and indole-containing tryptophan metabolites as well as four inflammatory markers measured at baseline were linked to incident all-cause (ACD) and Alzheimer’s disease dementia (AD) in binary outcomes and time-to-dementia analyses. Independent of several covariates, seven gut microbiome molecules, 5-hydroxyindole-3-acetic acid, indole-3-butyric acid, indole-3-acryloylglycine, indole-3-lactic acid, indole-3-acetic acid methyl ester, isobutyric acid, and 2-methylbutyric acid, but no inflammatory markers discriminated incident dementia cases from non-cases. Furthermore, 5-hydroxyindole-3-acetic acid (hazard ratio: 0.58; 0.36–0.94, *P* = 0.025) was associated with time-to-ACD. These molecules underpin gut microbiome-host interactions in the development of dementia and they may be crucial in its prevention and intervention strategies. Future larger epidemiological studies are needed to confirm our findings, specifically in exploring the repeatedly measured circulating levels of these molecules and investigating their causal relationship with dementia risk.

**Supplementary Information:**

The online version contains supplementary material available at 10.1007/s12035-023-03513-6.

## Introduction

Dementia is a neurodegenerative disease characterized by cognitive impairment that affects memory, cognitive abilities, and behavior, and significantly interferes with a person’s ability to perform daily activities [1]. The cognitive functional deterioration in dementia is beyond what is expected as the natural consequence of biological ageing [1]. Dementia has uncertain etiology, inherently complex pathophysiology, and heterogeneous manifestations [2]. Alzheimer’s disease dementia (AD) is the most frequent subtype, accounting for 60–80% of dementia as a whole, all-cause dementia (ACD) [3]. AD is driven by the brain accumulation of beta-amyloid plaques and tau protein tangles, but other mechanisms that include neuronal loss, synaptic dysfunction, neurodegeneration, and metabolic and inflammatory alterations might play earlier or more central role [4]. The global burden of dementia, influenced by the increasing life expectancy, demographics, and risk factors, was recently projected to increase in some countries such as the USA and Germany [5]. Indeed, a deeper insight into dementia’s well-known and emerging modifiable risk factors could have a major impact on its rising burden through targeted prevention and intervention strategies.

Interestingly, there is a growing recognition that the gut microbiome may play a role in the occurrence of dementia [6–8]. The major putative molecular mechanisms underlying this link are the production of gut microbiome bioactive molecules [6–9] and gut microbiome-induced systemic inflammation [6, 7, 10]. These gut microbiome bioactive molecules comprising those produced solely by the gut microbiome and those resulting from the host–gut microbiome co-metabolism are primarily bacterial endotoxins such as lipopolysaccharide (LPS), short-chain fatty acids (SCFA), and indole-containing tryptophan metabolites [6, 9, 11]. Crucially, some of these molecules are detectable in the systemic circulation [6, 9, 12] reaching concentrations up to and above those achieved by a typical drug dose [12] and they have been reported to cross the blood–brain barrier (BBB) [8, 9, 13, 14].

Remarkably, epidemiological studies have reported associations of gut microbiome molecules, specifically acetic acid (AA) and propionic acid (PA) [15], isobutyric acid and isovaleric acid [16] and indoxyl sulfate (IS) [17], LPS [18], LPS-binding protein (LBP) [19], AA [20], PA [21, 22], 2-methylbutyric acid, isovaleric acid, valeric acid, indole-3-pyruvic acid [22], and 5-hydroxyindole-3-acetic acid (5OH-IAA) [23, 24] with either ACD or AD risk but others did not observe associations with AA [16, 25], PA [16], and indole [16], LPS [22], indole-3-acetic acid (IAA) [23], indoleacrylic acid (IACR) [22], and 5OH-IAA [26]. Similarly, a few studies reported association of inflammatory markers, C-reactive protein (CRP), IL-1β, IL-6, and tumor necrosis factor-alpha (TNF-α) with either ACD or AD risk [27, 28], whereas others indicated no association with CRP [29–31], IL-1β [31], IL-6 [19, 31], and TNF-α [31]. These mixed findings may be partly due to the generally modest sample size of the aforementioned studies. Thus, larger studies are warranted. Furthermore, the levels of some gut microbiome molecules and systemic inflammatory markers as well as dementia risk are influenced by a myriad of factors such as body composition, smoking, alcohol consumption, and chronic diseases [2, 32–34]. These factors were not sufficiently accounted for in previous studies. Most important, the extensively reported association of some gut microbiome molecules and inflammatory markers [32, 33, 35–38] suggests that there is an intricate pathophysiological interplay between these molecules and inflammation. However, a joint investigation of these molecules and inflammatory markers in the context of dementia occurrence, which seems crucially important, has received limited attention.

To this end, the present epidemiological investigation sought to explore the circulating levels of selected gut microbiome molecules and inflammatory markers simultaneously and examine their association with the risk of ACD and AD while accounting for these important risk factors.

## Methods

### Study Population

The present study is embedded within the German study on Ageing, Cognition, and Dementia in Primary Care Patients (AgeCoDe) study. The AgeCoDe is a unique study in that it is a prospective cohort of elderly (≥75 years) general practitioner (GP) patients. Other inclusion criteria include being a native German language speaker or speaking German fluently, absence of severe hearing or vision impairments, and residency at home. The participants were recruited from six German cities, Bonn, Düsseldorf, Hamburg, Leipzig, Mannheim, and Munich. The study commenced between 2003 and 2004, and detailed clinical information that includes standardized cognitive testing was conducted. Follow-up assessments were performed every 18 months and every 10 months after follow-up seven. At follow-up three (henceforth, baseline) which commenced in 2007, participants provided blood samples at the GP’s office, from where the samples were transported to the central laboratory for storage. For the current study, we considered incident dementia, that is, dementia diagnosis between baseline and follow-up nine (henceforth, end of follow-up), which was completed in 2016. The ethical approval for the AgeCoDe study was obtained from the Ethics Commission of the University of Bonn 050/02, 258/07; the Ethics Commission of the Medical Faculty of the Heinrich Heine University Düsseldorf 2079/2002, 2999/2008; the Ethics Commission of the Medical Association Hamburg OB/08/02, 2817/2007; the Ethics Commission at the Medical Center of the University of Leipzig 143/2002, 309/2007. The ethical approval for the present biomarker analysis was obtained from the Ethics Commission of the University of Bonn 245/22. All participants gave written informed consent. Details of the recruitment of participants and assessment of dementia in the AgeCoDe have been reported previously [39].

### Study Design

The eligible individuals were those free of dementia at baseline and with information on classical risk factors of dementia, namely age, sex, and body mass index (BMI) at baseline. Consequently, the total size of this current full cohort was *N* = 1323, of which 281 (21%) developed incident ACD. Incident dementia was defined as cases from the full cohort occurring between baseline and end. Person time (time-to-event) was calculated from the baseline date to the date of diagnosis of dementia, or the end of study, whichever occurred first. We censored individuals at the end of the study on 29 November 2016.

From these *N* = 1323 eligible individuals, we designed a classical case–cohort study that comprise 50% (*N* = 662) subcohort selected via a simple random sampling without replacement and all the ACD cases outside the subcohort. The sampling fraction and method were chosen for their reported efficiency [40]. Consequently, the case–cohort sample for ACD analyses was *N* = 805 (*N* = 143 non-subcohort cases, *N* = 138 subcohort cases, and *N* = 524 subcohort non-cases). Secondarily, we selected AD cases outside the subcohort to form an AD case–cohort sample of *N* = 740 (*N* = 78 non-subcohort cases, *N* = 73 subcohort cases, and *N* = 589 subcohort non-cases).

### Measurement of Gut Microbiome Molecules and Inflammatory Markers

All biomarkers were measured from EDTA plasma collected at baseline. All laboratory analyses were blinded to the participants’ dementia status and any characteristics.

#### Lipopolysaccharide and Lipopolysaccharide-Binding Protein

LPS was measured by a quantitative sandwich enzyme immunoassay technique (MBS702450; MyBiosource, San Diego, CA, USA) and LBP by a solid-phase, two-site chemiluminescent immunometric assay (IMMULITE®1000, Siemens Healthcare GmbH, Erlangen, Germany). Further processing of the samples was carried out according to the specifications from the kit instructions or according to the specifications of the laboratories. For both LPS and LBP, the intra-assay coefficient of variation (CV) was <8% and inter-assay CV was <10%.

#### Indole-Containing Tryptophan Metabolites

Targeted metabolomics quantification of the concentrations 9 indole-containing tryptophan metabolites, IAA, indole-3-acetic acid methyl ester (IAA ME), 5OH-IAA, indole-3-propionic acid (IPA), indole-3-butyric acid (IBA), indole-3-lactic acid (ILA), indole-3-carboxaldehyde (ICARB), indole-3-acryloylglycine (IAG), IS was determined by ultra high performance liquid chromatography-electrospray ionization-triple quadrupole-mass spectrometry, as reported previously [41]. Other metabolites, which include tryptophan, methionine, tyrosine, serotonin, and N-acetyl-tryptophan, were also quantified in this targeted assay. The intra-day CV for all metabolites in the present analysis was less than 15%.

#### Short-Chain Fatty Acids

The measurement of the SCFA was according to previously reported targeted metabolomics analysis [42]. We measured eight SCFA namely AA, PA, isobutyric acid, butyric acid (BA), 2-methylbutyric acid, isovaleric acid, valeric acid (VA), and hexanoic acid (HA). The intra-day CV for all SCFAs was less than 20%.

#### Inflammatory Markers

The multiplexing analysis of IL-1β, IL-6, and TNFα was performed using the Luminex™ 200 system (Luminex, Austin, TX, USA). The intra-assay CV for all three markers was less than 10%. The inter-assay CV for IL-1β and IL-6 was less than 15% while TNF-α was less than 20%. High-sensitivity CRP was measured at the central laboratory of the University Hospital in Bonn.

### Assessment of Covariates: Sociodemographic, Anthropometry, Lifestyle Factors, and Prevalent Diseases

All covariates were assessed at baseline. Standardized questionnaires were used to obtain information on age, sex, weight, height, education, last employment status, smoking status, alcohol consumption, physical activity, social status (marital status and living alone), and the presence of prevalent disease (hypertension, type 2 diabetes, coronary heart disease, stroke, depression, hearing and visual impairment, and traumatic brain injury). BMI was calculated as weight in kilograms divided by the squared height in meters. Educational level was categorized into low (inadequately completed or elementary schooling), middle (secondary), and high (tertiary). Participants whose last employment status was manual jobs, salaried jobs, civil service jobs, and self-employed were categorized as employed and others were categorized as unemployed. Smoking was assessed as the current smoking of cigarettes, a pipe, cigars, or other tobacco products. Individuals who were current non-smokers but smoked for any number of years of smoking were categorized as ex-smokers. Alcohol consumption was determined from the frequency and quantity of consumption and converted to a uniform measure of grams per day. Physically active individuals were those who had more than one per week cycling, hiking or long walks, swimming, gymnastics, and other sports such as golf. Marital status was categorized into four groups, widowed, divorced, married, and single. The Mini-Mental State Examination (MMSE) score was used to assess global cognitive function at baseline. Depression was assessed based on the 15-item version of the Geriatric Depression Scale dichotomized into <6 points (no evidence of depressive symptoms) and ≥6 points (evidence of depressive symptoms). Medication use was obtained from questionnaire. Plasma hemoglobin A1c (HbA1c) was measured using the Roche/Hitachi-ModularSystems (Roche) according to the manufacturer’s protocols. Apolipoprotein E (APOE)-ɛ4) status was determined from leucocyte DNA.

### Outcomes

Dementia diagnoses were based on a validated, structured interview for the diagnosis of dementia of the Alzheimer type, multi-infarct (or vascular) dementia, and dementias of other etiology according to the Diagnostic and Statistical Manual of Mental Disorders (DSM)-III-R, DSM-IV, and ICD-10 (Structured Interview for Diagnosis of Dementia of Alzheimer type, Multi-infarct Dementia and Dementia of other etiology according to DSM-IV and ICD-10 (SIDAM) [43]), implemented by trained research assistants. The SIDAM consists of a cognitive test battery (55 items including the Mini-Mental State Examination and covering the cognitive domains of orientation, memory, abstract reasoning, verbal ability and calculation, constructional ability, aphasia and apraxia) and a section for clinical diagnostic impression and rating of psychosocial impairment with a scale for the assessment of activities of daily living. Dementia was diagnosed according to the criteria of the DSM-IV, which comprise a diagnostic algorithm in the SIDAM including cognitive impairment on the SISCO score and impairment in ADL (score of ≥2 on the SIDAM ADL Scale). The diagnosis of dementia in AD was established according to the National Institute of Neurological and Communicative Disorders and Stroke–Alzheimer’s Disease and Related Disorders Association criteria for probable AD [44]. Vascular dementia diagnosis was guided by the National Institute of Neurological Disorders and Stroke–Association Internationale pour la Recherché et l’Enseignement en Neurosciences criteria [45] (i.e., evidence of a cerebrovascular event [Hashinski-Rosen Scale and medical history] and temporal association of the cerebrovascular event with cognitive decline). Mixed dementia was diagnosed in the absence of temporal association of the cerebrovascular event with cognitive decline. For all analyses, individuals with mixed dementia and dementia in AD were combined. Dementia diagnosis in individuals who were not personally interviewed was based on the Global Deterioration Scale and the Blessed Dementia Scale subscales. A score of at least 4 on the Global Deterioration Scale represented a diagnosis of dementia. The diagnosis was established in these cases if the causal information provided was sufficient for judgment using the aforementioned criteria. All diagnoses were made in consensus conferences that included the interviewer and experienced geriatric psychiatrists or geriatricians. The primary and secondary outcomes for the present study were ACD and AD, respectively.

## Statistical Analysis

### Descriptive Analysis

Analysis was performed for ACD (*n* = 805) and AD (*n* = 740) case–cohorts separately. Continuous and categorical variables were summarized as median (25% and 75% percentile), and count (percentage), respectively. Difference in continuous and categorical predictors across dementia status were tested with the Kruskal–Wallis rank-sum test and either Pearson’s Chi-squared or Fisher’s exact test, respectively.

### Multivariable Modeling of the Association of Gut Microbiome Molecules and Inflammatory Markers with Dementia Risk

#### Selection of Covariates

We identified covariates for the causal inference between gut microbiome molecules and inflammatory markers with dementia risk using a directed acyclic graph (DAG). Based on a priori knowledge and biological plausibility, we used a DAG to draw the directions of the paths between the gut microbiome molecules or inflammatory markers and covariates, between covariates and dementia, and between covariates. A minimal sufficient adjustment set of confounders was selected and used as covariates.

From the DAG, our covariates were as follows: age (years), sex (men and women: reference), BMI (kg/m^2^), APOE-ɛ4 (homozygous, heterozygous, absent: reference), smoking status (smokers, ex-smokers, non-smokers: reference), alcohol intake (g/day), educational level (high, middle low: reference), employment (employed and unemployed: reference), physical activity (active and inactive: reference), marital status (widowed, divorced, married, single: reference), living alone (yes and no: reference), family history of dementia (yes and no: reference), prevalent hypertension, diabetes mellitus, coronary heart disease, stroke, depression, traumatic brain injury (TBI) (yes and no: reference), hearing impairment (significant hearing loss, mild hearing loss, and no impairment: reference), visual impairment (most severe visual impairment, considerable visual impairment, difficult vision, and no impairment: reference), mediation (yes and no: reference), study center (Leipzig, Hamburg, Düsseldorf, Mannheim Munich, Bonn: reference), MMSE score, HbA1c, and habitual diet. Due to unavailability of self-reported dietary intake data in this study and since plasma concentrations of tryptophan, methionine, and tyrosine have been reported to differentiate habitual diet groups, fish-eaters and vegetarians, meat-eaters, and vegans [46], we used them as proxies for habitual diet. We additionally adjusted for serotonin and N-acetyl-tryptophan. The 23 primary predictors (molecules and inflammatory markers) were LPS (pg/ml), LBP (μg/ml), IAA, IAA ME, 5OH-IAA, IPA, IBA, ILA, ICARB, IAG, IS, AA, PA, isobutyric acid, BA, 2-methylbutyric acid, isovaleric acid, VA, and HA (μM), CRP (mg/ml), and IL-1β, IL-6, and TNF-α (pg/ml). We checked the bivariate association between the primary predictors and covariates using the Spearman correlation and Kruskal–Wallis rank-sum test.

#### Handling of Missingness

We examined the proportion of missingness across the variables. Globally, we evaluated the missing completely at random (MCAR) assumption with Little’s MCAR test. Afterwards, we tested the missing data mechanism of each variable with a regression-based approach. We imputed the missing values when MCAR or missing at random (MAR) assumption is reasonable.

#### Statistical Power Analysis

There is no well-established power calculation for case–cohort studies with continuous predictors and non-rare events. Therefore, we used the standard power method for case–control studies. For this method, we used type I error rate of 0.05 and the values of three predictors of AD risk of André et al. [19]. Since the variables were standardized beforehand, we assumed them to be normally distributed with 0 mean and a standard deviation of 1. The dementia-associated adjusted odds ratio of the main predictor was 1.3 and the two covariates were 1.09 and 0.98. We independently included sample size and proportion of events for the ACD and AD. The power of the *n* = 805 ACD case–cohort was 91% and for the *n* = 740 AD case–cohort was 76%. Indeed, a sample size of *n* = 577 and *n* = 809 would produce the conventionally acceptable power (≥ 80%) for the ACD and AD case–cohorts, respectively. Considering that this standard power method has been shown to produce conservative estimates for case–cohort studies with non-rare events and binary predictors [47], it is likely that the actual power of our study samples would be higher.

##### Multivariable Regression Analysis

Continuous and categorical predictors were standardized and dummy coded, respectively. To estimate the association of the primary predictors (gut microbiome molecules and inflammatory markers) adjusted for covariates with ACD and AD risk, we adopted one of the recommended multivariable statistical approaches for analyzing case–cohort data, which comprises an initial variable selection step in binary outcome analysis with logistic regression followed by time-to-event analysis with weighted Cox proportional hazards (Cox PH) regression [48]. In the current variable selection step, we used three methods, the parametric adaptive Least Absolute Shrinkage and Selection Operator (aLASSO) logistic regression with 10-fold cross-validation, the random forest–based Boruta algorithm (RF-Boruta), and the recursive feature elimination implemented using Naïve Bayes algorithm (RFE-NB) with 10-fold cross-validation. These methods help to capture all (linear, non-linear, and interactions) complex inter-relationships among the predictors so that those that robustly discriminate dementia cases from non-cases are recovered. It also reduces potentially exaggerating the strength of associations of the multiple primary predictors with dementia risk and over-adjustment for covariates in the time-to-dementia models. Predictors with non-zero coefficients from the aLASSO logistic regression, predictors confirmed as important from the RF-Boruta, and optimal features from the RFE-NB were considered as the true relevant predictors of dementia. These true relevant predictors were used to estimate time-to-ACD and time-to-AD. We fitted Self-Prentice weighted Cox PH and checked for the PH assumption. The continuous predictors that violated the PH assumption were included with their time-varying form, while categorical predictors were handled by stratification of their baseline hazard function leading to no estimates for them. In addition, we fitted the accelerated failure time (AFT) models with weighted least-squares approach.

#### Bias Analysis

If any main predictor is significantly associated with time-to-dementia in the Cox PH, we estimated the strength of association (*E*-value) [49] on the risk ratio scale that unmeasured confounder(s) would need to have with both the predictor and dementia risk to completely explain away the predictor–dementia risk association, conditional on the measured covariates. Furthermore, by excluding early (first year of follow-up) incident cases, we addressed reverse causality in which the long prodromal phase and premorbid (subclinical) dementia might have directly altered the levels of these biomarkers rather than the reverse.

All probabilities were two-sided and significant level was set at *P* < 0.05. Statistical analyses were performed using R version 4.2.1.

## Results

### Descriptive Analysis

Tables 1 and 2 summarize the baseline characteristics of the *n* = 805 ACD and *n* = 740 AD study populations with 281 (35%) cases and 151 (20%) cases, respectively. About two-thirds were women with median age and BMI of 83 years and 25 kg/m^2^, respectively. Around 77% were physically active, about 5% were current smokers, mild alcohol consumers, and had normal MMSE score. Cases for both dementia outcomes were more likely to be women, older, more likely to be APOE-ɛ4 carriers, less physically active, less likely to be married, more likely to live alone, and had lower MMSE score. AD cases also had lower BMI. In addition, cases had higher IBA and lower 5OH-IAA (Table 3).

**Table 1:** Baseline characteristics of the all-cause dementia case-cohort

	All-cause dementia case-cohort (subcohort: *n* = 662; cases outside subcohort: *n* = 143)			*P*^‡^
	Total (*n* = 805)	Cases (*n* = 281)	Non-cases (*n* = 524)	
Age, years*	83 (81, 86)	84 (82, 87)	83 (81, 86)	<0.001
Women^†^	534 (66.3%)	212 (75.4%)	322 (61.5%)	<0.001
Body mass index*	25.39 (23.15, 28.13)	24.92 (22.86, 28.40)	25.83 (23.34, 28.04)	0.106
Apolipoprotein E-ε4 status^†^				0.004
Absent	629 (81.5%)	201 (75.0%)	428 (84.9%)	
Heterozygous	140 (18.1%)	65 (24.3%)	75 (14.9%)	
Homozygous	3 (0.4%)	2 (0.7%)	1 (0.2%)	
Family history of dementia^†^	198 (24.6%)	79 (28.1%)	119 (22.7%)	0.09
Educational level^†^				0.405
Low	482 (59.9%)	173 (61.6%)	309 (59.0%)	
Middle	229 (28.4%)	81 (28.8%)	148 (28.2%)	
High	94 (11.7%)	27 (9.6%)	67 (12.8%)	
Employment history, Unemployed^†^	208 (25.8%)	80 (28.5%)	128 (24.4%)	0.212
Smoking status^†^				0.993
Non-smokers	518 (64.4%)	181 (64.6%)	337 (64.3%)	
Ex-smokers	245 (30.5%)	85 (30.4%)	160 (30.5%)	
Smokers	41 (5.1%)	14 (5.0%)	27 (5.2%)	
Alcohol consumption, g/day*	2.57 (0, 10)	0 (0, 10)	3.43 (0, 10)	0.124
Physically active^†^	621 (77.1%)	199 (70.8%)	422 (80.5%)	0.002
Marital status^†^				0.01
Single	57 (7.1%)	22 (7.9%)	35 (6.7%)	
Married	287 (35.7%)	79 (28.2%)	208 (39.7%)	
Divorced	35 (4.4%)	11 (3.9%)	24 (4.6%)	
Widowed	425 (52.9%)	168 (60.0%)	257 (49.0%)	
Living alone^†^	430 (53.4%)	164 (58.4%)	266 (50.8%)	0.039
Prevalent hypertension^†^	633 (81.4%)	220 (81.2%)	413 (81.5%)	0.924
Prevalent diabetes^†^	201 (25.9%)	72 (26.7%)	129 (25.5%)	0.722
Hemoglobin A1c, %*	5.70 (5.50, 6.00)	5.70 (5.50, 6.00)	5.70 (5.50, 6.00)	0.638
Prevalent coronary heart disease^†^	271 (35.0%)	89 (32.8%)	182 (36.1%)	0.363
Prevalent stroke^†^	52 (6.7%)	22 (8.1%)	30 (5.9%)	0.244
Prevalent depression^†^	166 (21.3%)	66 (24.4%)	100 (19.7%)	0.133
Prevalent visual loss^†^				0.447
No impairment	635 (78.9%)	214 (76.2%)	421 (80.3%)	
Difficult vision	121 (15.0%)	50 (17.8%)	71 (13.5%)	
Considerable visual impairment	39 (4.8%)	14 (5.0%)	25 (4.8%)	
Most severe visual impairment	10 (1.2%)	3 (1.1%)	7 (1.3%)	
Prevalent hearing loss^†^				0.714
No impairment	458 (56.9%)	156 (55.5%)	302 (57.6%)	
Mild hearing loss	324 (40.2%)	118 (42.0%)	206 (39.3%)	
Significant hearing loss	23 (2.9%)	7 (2.5%)	16 (3.1%)	
Traumatic brain injury†	6 (0.8%)	4 (1.5%)	2 (0.4%)	0.307
Mini-Mental State Examination score*	28.00 (27.00, 29.00)	28.00 (26.96, 29.00)	29.00 (27.39, 30.00)	<0.001
Methionine*	9.46 (7.74, 11.45)	9.38 (7.86, 11.35)	9.53 (7.66, 11.54)	0.672
Tryptophan*	32.55 (28.23, 37.48)	32.33 (27.72, 37.83)	32.64 (28.35, 37.42)	0.659
Tyrosine*	52.52 (45.63, 60.99)	51.62 (44.95, 61.33)	53.03 (46.15, 60.88)	0.338
Serotonin*	0.13 (0.06, 0.24)	0.13 (0.06, 0.24)	0.13 (0.06, 0.24)	0.819
*N*-acetyl -tryptophan*	0.04 (0.04, 0.05)	0.04 (0.04, 0.05)	0.04 (0.04, 0.05)	0.413
Medication use^†^	795 (98.8%)	277 (98.6%)	518 (98.9%)	0.746
Study center^†^				0.492
Leipzig	160 (19.9%)	48 (17.1%)	112 (21.4%)	
Hamburg	121 (15.0%)	43 (15.3%)	78 (14.9%)	
Düsseldorf	120 (14.9%)	37 (13.2%)	83 (15.8%)	
Mannheim	137 (17.0%)	50 (17.8%)	87 (16.6%)	
Bonn	119 (14.8%)	47 (16.7%)	72 (13.7%)	
Munich	148 (18.4%)	56 (19.9%)	92 (17.6%)	
Follow-up, years*	5.67 (3.34, 7.51)	4.61 (3.21, 6.02)	6.84 (3.65, 7.70)	<0.001

*Median (25% and 75% percentile)

^†^Count (percentage)

^‡^*P* value obtained from Kruskal–Wallis rank-sum test and either Pearson’s Chi-squared or Fisher’s exact test

**Table 2:** Baseline characteristics of the Alzheimer’s disease dementia case-cohort

	Alzheimer’s disease dementia case-cohort (subcohort: *n* = 662; cases outside subcohort: *n* = 78)			*P*^‡^
	Total (*n* = 740)	Cases (*n* = 151)	Non-cases (*n* = 589)	
Age, years*	83 (81, 86)	85 (83, 87)	83 (81, 86)	<0.001
Women^†^	484 (65.4%)	116 (76.8%)	368 (62.5%)	<0.001
Body mass index*	25.39 (23.15, 28.01)	24.68 (22.59, 27.68)	25.71 (23.31, 28.06)	0.021
Apolipoprotein E-ε4 status^†^				0.002
Absent	583 (82.1%)	103 (72.0%)	480 (84.7%)	
Heterozygous	125 (17.6%)	39 (27.3%)	86 (15.2%)	
Homozygous	2 (0.3%)	1 (0.7%)	1 (0.2%)	
Family history of dementia^†^	183 (24.7%)	43 (28.5%)	140 (23.8%)	0.232
Educational level^†^				0.49
Low	443 (59.9%)	92 (60.9%)	351 (59.6%)	
Middle	208 (28.1%)	45 (29.8%)	163 (27.7%)	
High	89 (12.0%)	14 (9.3%)	75 (12.7%)	
Employment history, unemployed^†^	189 (25.5%)	42 (27.8%)	147 (25.0%)	0.473
Smoking status^†^				0.902
Non-smokers	478 (64.7%)	99 (66.0%)	379 (64.3%)	
Ex-smokers	223 (30.2%)	43 (28.7%)	180 (30.6%)	
Smokers	38 (5.1%)	8 (5.3%)	30 (5.1%)	
Alcohol consumption, g/day*	3.20 (0, 10)	0 (0, 10)	3.43 (0, 10)	0.164
Physically active^†^	575 (77.7%)	103 (68.2%)	472 (80.1%)	0.002
Marital status^†^				0.001
Single	53 (7.2%)	11 (7.3%)	42 (7.1%)	
Married	270 (36.5%)	34 (22.5%)	236 (40.1%)	
Divorced	33 (4.5%)	7 (4.6%)	26 (4.4%)	
Widowed	383 (51.8%)	99 (65.6%)	284 (48.3%)	
Living alone^†^	388 (52.4%)	94 (62.3%)	294 (49.9%)	0.007
Prevalent hypertension^†^	581 (81.0%)	115 (78.2%)	466 (81.8%)	0.331
Prevalent diabetes^†^	181 (25.3%)	40 (27.2%)	141 (24.8%)	0.553
Hemoglobin A1c, %*	5.70 (5.50, 6.00)	5.70 (5.50, 6.00)	5.70 (5.50, 6.00)	0.424
Prevalent coronary heart disease^†^	252 (35.3%)	50 (34.0%)	202 (35.6%)	0.715
Prevalent stroke^†^	44 (6.1%)	6 (4.1%)	38 (6.7%)	0.243
Prevalent depression^†^	152 (21.2%)	33 (22.4%)	119 (20.9%)	0.678
Prevalent visual loss^†^				0.909
No impairment	586 (79.2%)	118 (78.1%)	468 (79.5%)	
Difficult vision	107 (14.5%)	22 (14.6%)	85 (14.4%)	
Considerable visual impairment	37 (5.0%)	9 (6.0%)	28 (4.8%)	
Most severe visual impairment	10 (1.4%)	2 (1.3%)	8 (1.4%)	
Prevalent hearing loss^†^				0.914
No impairment	417 (56.4%)	87 (57.6%)	330 (56.0%)	
Mild hearing loss	300 (40.5%)	59 (39.1%)	241 (40.9%)	
Significant hearing loss	23 (3.1%)	5 (3.3%)	18 (3.1%)	
Traumatic brain injury†	5 (0.7%)	1 (0.7%)	4 (0.7%)	0.918
Mini-Mental State Examination score*	28.91 (27.00, 29.00)	28.00 (26.00, 29.00)	29.00 (27.00, 29.25)	< 0.001
Methionine*	9.52 (7.74, 11.47)	9.23 (7.71, 11.23)	9.57 (7.75, 11.52)	0.413
Tryptophan*	32.56 (28.16, 37.49)	31.97 (26.80, 37.26)	32.74 (28.35, 37.49)	0.274
Tyrosine*	52.44 (45.68, 61.02)	51.78 (44.29, 62.03)	52.66 (46.06, 60.83)	0.423
Serotonin*	0.13 (0.06, 0.23)	0.13 (0.06, 0.22)	0.13 (0.06, 0.25)	0.46
*N*-acetyl -tryptophan*	0.04 (0.04, 0.05)	0.04 (0.04, 0.05)	0.04 (0.04, 0.05)	0.541
Medication use^†^	730 (98.6%)	150 (99.3%)	580 (98.5%)	0.6961
Study center^†^				0.054
Leipzig	148 (20.0%)	24 (15.9%)	124 (21.1%)	
Hamburg	113 (15.3%)	24 (15.9%)	89 (15.1%)	
Düsseldorf	102 (13.8%)	13 (8.6%)	89 (15.1%)	
Mannheim	128 (17.3%)	26 (17.2%)	102 (17.3%)	
Bonn	107 (14.5%)	24 (15.9%)	83 (14.1%)	
Munich	142 (19.2%)	40 (26.5%)	102 (17.3%)	
Follow-up, years*	5.83 (3.33, 7.55)	4.36 (3.26, 5.90)	6.48 (3.43, 7.66)	< 0.001

*Median (25% and 75% percentile)

^†^Count (percentage)

^‡^*P* value obtained from Kruskal–Wallis rank-sum test and either Pearson’s Chi-squared or Fisher’s exact test

**Table 3:** Description of the primary predictors

	All-cause dementia case-cohort (subcohort: *n* = 662; cases outside subcohort: *n* = 143)			*P*^‡^	Alzheimer’s disease dementia case-cohort (subcohort: *n* = 662; cases outside subcohort: *n* = 78)			*P*^‡^
	Total (*n* = 805)	Cases (*n* = 281)	Non-cases (*n* = 524)		Total (*n* = 740)	Cases *(n* = 151)	Non-cases (*n* = 589)	
Lipopolysaccharide*	107.91 (69.49, 168.92)	111.45 (71.94, 174.83)	104.78 (65.85, 164.82)	0.112	106.86 (69.07, 165.10)	112.76 (71.94, 173.14)	105.09 (68.09, 164.65)	0.224
LPS-binding protein^#^	5.20 (4.10, 6.30)	5.20 (4.10, 6.50)	5.20 (4.10, 6.30)	0.824	5.20 (4.10, 6.30)	5.30 (4.00, 6.30)	5.20 (4.10, 6.30)	0.78
Indole-3-acetic acid^†^	5.12 (3.93, 7.15)	5.33 (4.12, 7.37)	5.05 (3.79, 7.08)	0.094	5.09 (3.90, 7.15)	5.12 (4.08, 7.61)	5.07 (3.84, 7.08)	0.363
Indole-3-acetic acid methyl ester^†^	0.01 (0.01, 0.01)	0.01 (0.01, 0.01)	0.01 (0.01, 0.01)	0.742	0.01 (0.01, 0.01)	0.01 (0.01, 0.01)	0.01 (0.01, 0.01)	0.952
Indole-3-propionic acid^†^	5.13 (3.09, 7.70)	4.93 (2.83, 7.95)	5.17 (3.19, 7.32)	0.669	5.01 (3.08, 7.41)	4.86 (2.95, 7.70)	5.145 3.132, 7.334)	0.426
Indole-3-butyric acid^†^	0.03 (0.02, 0.04)	0.03 (0.02, 0.04)	0.03 (0.02, 0.04)	0.003	0.03 (0.02, 0.04)	0.03 (0.02, 0.04)	0.03 (0.02, 0.04)	0.033
Indole-3-lactic acid^†^	1.45 (1.13, 1.87)	1.39 (1.12, 1.77)	1.49 (1.13, 1.90)	0.099	1.45 (1.12, 1.87)	1.40 (1.11, 1.81)	1.46 (1.12, 1.87)	0.449
Indole-3-carboxaldehyde^†^	0.27 (0.22, 0.39)	0.27 (0.23, 0.39)	0.27 (0.22, 0.40)	0.882	0.27 (0.22, 0.39)	0.27 (0.23, 0.38)	0.27 (0.22, 0.40)	0.406
Indole-3-acryloylglycine^†^	0.04 (0.03, 0.07)	0.04 (0.02, 0.07)	0.04 (0.03, 0.07)	0.711	0.04 (0.03, 0.06)	0.04 (0.03, 0.06)	0.04 (0.03, 0.07)	0.962
Indoxyl sulfate^†^	34.57 (20.96, 51.74)	34.75 (22.06, 51.08)	34.41 (20.80, 51.96)	0.82	34.58 (20.98, 51.77)	35.12 (22.31, 50.24)	34.31 (20.84, 52.00)	0.841
5-hydroxyindole-3-acetic acid^†^	0.77 (0.48, 1.22)	0.68 (0.47, 1.10)	0.82 (0.50, 1.29)	0.003	0.79 (0.48, 1.25)	0.67 (0.46, 1.12)	0.82 (0.50, 1.29)	0.014
Acetic acid^†^	22.72 (17.92, 30.25)	22.69 (17.39, 30.27)	22.77 (18.19, 30.14)	0.461	22.74 (18.06, 30.02)	22.94 (17.52, 29.17)	22.62 (18.12, 30.30)	0.884
Propionic acid^†^	3.51 (2.83, 4.39)	3.49 (2.87, 4.31)	3.53 (2.81, 4.42)	0.866	3.53 (2.86, 4.42)	3.56 (3.08, 4.42)	3.52 (2.81, 4.42)	0.292
Isobutyric acid^†^	0.47 (0.37, 0.65)	0.47 (0.36, 0.64)	0.47 (0.37, 0.65)	0.789	0.47 (0.37, 0.65)	0.46 (0.37, 0.65)	0.47 (0.37, 0.65)	0.677
Butyric acid^†^	0.94 (0.76, 1.21)	0.96 (0.76, 1.15)	0.93 (0.76, 1.24)	0.662	0.94 (0.76, 1.21)	0.97 (0.76, 1.14)	0.94 (0.76, 1.23)	0.464
2-methylbutyric acid^†^	0.36 (0.28, 0.48)	0.37 (0.28, 0.49)	0.36 (0.28, 0.48)	0.797	0.36 (0.28, 0.48)	0.37 (0.28, 0.50)	0.36 (0.28, 0.48)	0.852
Isovaleric acid^†^	0.42 (0.30, 0.63)	0.42 (0.29, 0.63)	0.42 (0.30, 0.62)	0.571	0.42 (0.29, 0.62)	0.41 (0.27, 0.58)	0.43 (0.30, 0.63)	0.253
Valeric acid^†^	0.24 (0.19, 0.32)	0.24 (0.19, 0.31)	0.25 (0.19, 0.33)	0.514	0.25 (0.19, 0.32)	0.24 (0.18, 0.32)	0.25 (0.19, 0.33)	0.369
Hexanoic acid^†^	1.49 (1.15, 1.91)	1.44 (1.13, 1.84)	1.53 (1.17, 1.95)	0.058	1.51 (1.17, 1.94)	1.51 (1.15, 1.94)	1.51 (1.17, 1.93)	0.813
CRP, mg/mL	2.15 (1.11, 4.32)	2.14 (1.04, 4.14)	2.16 (1.18, 4.50)	0.349	2.14 (1.12, 4.38)	1.92 (1.01, 4.31)	2.19 (1.19, 4.47)	0.158
Interleukin (IL)-1β^#^	3.33 (1.38, 9.51)	3.28 (1.13, 8.95)	3.33 (1.47, 9.76)	0.393	3.28 (1.36, 9.30)	3.19 (1.13, 8.92)	3.28 (1.38, 9.55)	0.651
IL-6^#^	3.84 (2.30, 6.84)	3.88 (2.42, 6.79)	3.77 (2.25, 6.97)	0.68	3.84 (2.30, 7.03)	4.01 (2.38, 7.09)	3.75 (2.30, 7.03)	0.672
Tumor necrosis factor-α^#^	5.59 (4.23, 7.26)	5.54 (4.00, 6.96)	5.61 (4.27, 7.42)	0.212	5.61 (4.23, 7.35)	5.66 (4.09, 7.43)	5.59 (4.25, 7.33)	0.92

All values are in median (25% and 75% percentile), *pg/mL, ^#^μg/mL, ^†^μM, ^*‡*^*P* value obtained from Kruskal–Wallis rank-sum test

### Multivariable Modeling of the Association of Gut Microbiome Molecules and Inflammatory Markers with Dementia Risk

Some primary predictors are intercorrelated, highest between IAG and IPA, and between 2-methylbutyric acid with isobutyric acid and isovaleric acid (Table A.1). Furthermore, at least one primary predictor was associated with a covariate, with the largest proportion of the primary predictors associated with tryptophan and study center (Table A.1 and Table A.2).

### Handling of Missingness

The proportion of missingness was low to moderate, between 0.1 and 6.8% in the ACD case–cohort and between 0.1 and 7.2% in the AD casecohort (Table A.3). For the ACD case–cohort, VA, PA, BA, HA, marital status, and smoking status were MAR, while other variables were MCAR. For the AD case–cohort, VA, BA, marital status, and smoking status were MAR, while other variables were MCAR (Table A.3). Although the global MCAR assumption was not significant for both datasets (*P* = <0.001), the fact that the predictors were a mix of MCAR and MAR suggests that the MAR assumption is optimally appropriate for both datasets. Hence, missing values were imputed using the non-parametric multivariate imputation by the chained random forest weighted by the number of non-missing values per observation.

### Multivariable Regression Analysis

#### All-Cause Dementia

**Fig. 1:**
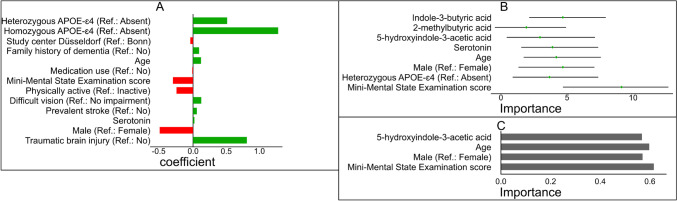
The true relevant predictors of all-cause dementia risk obtained from adaptive Least Absolute Shrinkage and Selection Operator logistic regression (green: positively associated predictors, red: negatively associated predictors) (**A**), random forest–based Boruta algorithm (Importance = median, minimum, and maximum *z*-scores of importance computed over multiple iterations) (**B**), and recursive feature elimination implemented using Naïve Bayes algorithm (Importance = area under the ROC curve (AUC) score importance) (**C**)

The 13 (eight positive and five negative) non-zero predictors from the aLASSO logistic regression were age, sex, heterozygous APOE-ɛ4 vs. none, homozygous APOE-ɛ4 vs none, MMSE score, family history of dementia, physically active vs. inactive, prevalent visual impairment, prevalent stroke, TBI, medication use, serotonin, and study center Düsseldorf vs. Bonn (Fig. 1A). The RF-Boruta confirmed eight important predictors, 5OH-IAA, IBA, 2-methylbutyric acid, age, sex, MMSE, heterozygous APOE-ɛ4 vs. none, and serotonin (Fig. 1B). The RFE-NB showed that a four-predictor model with age, sex, MMSE score, and 5OH-IAA optimally discriminates ACD cases from non-cases (Fig. 1C). Overall, 16 true relevant predictors discriminated ACD cases from non-cases. These were three gut microbiome molecules (5OH-IAA, IBA, 2-methylbutyric acid) and 13 covariates (age, sex, homozygous APOE-ɛ4 vs none, heterozygous APOE-ɛ4 vs. none, MMSE score, family history of dementia, physical active vs. inactive, prevalent visual impairment, prevalent stroke, history of TBI, medication, serotonin, and study center Düsseldorf vs. Bonn).

**Fig. 2:**
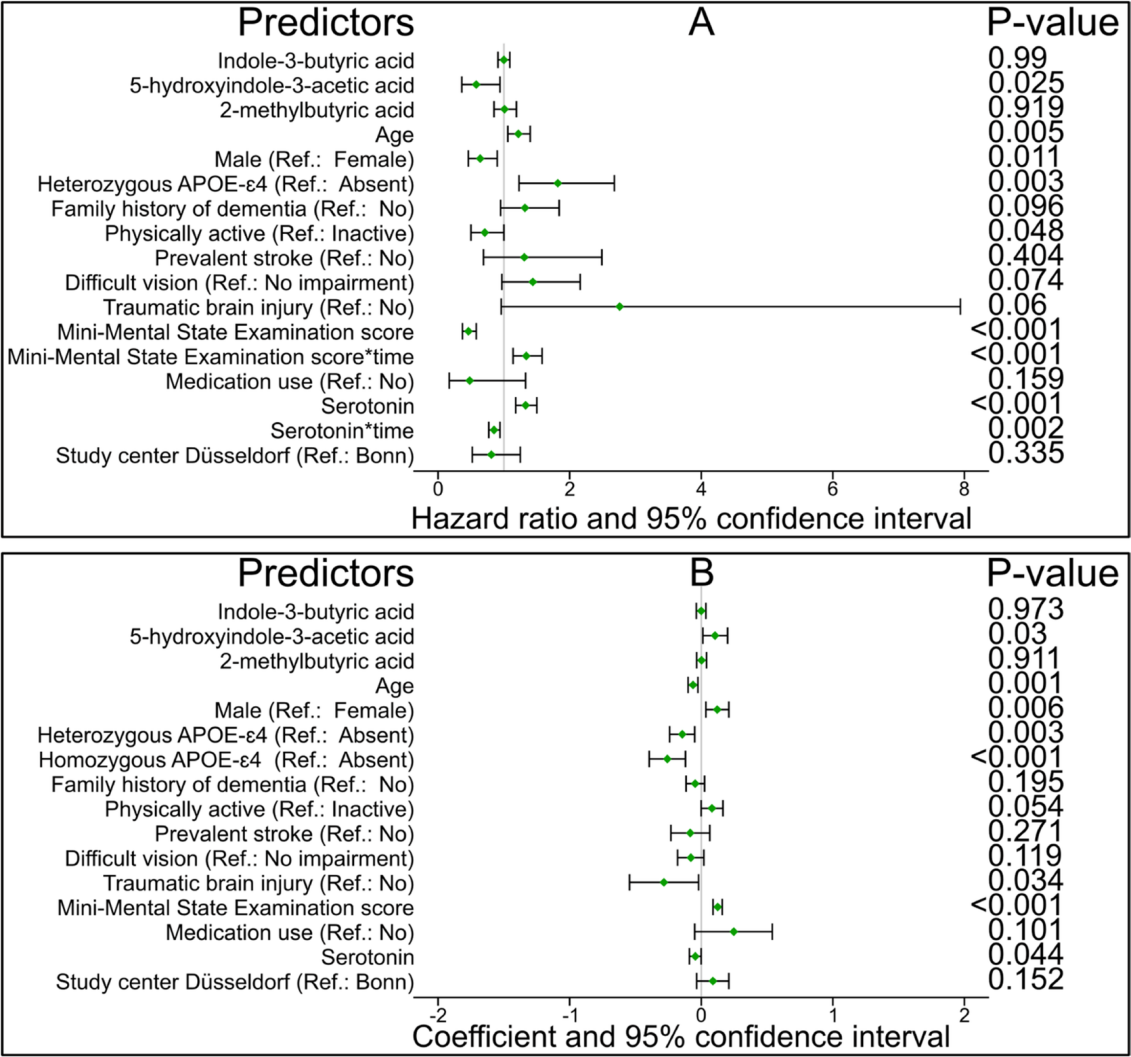
Association between true relevant predictors and time-to-all cause dementia risk. **(A)** Weighted Cox proportional hazard model (hazard ratio and 95% confidence interval). **(B**) Weighted least-squares accelerated failure time (coefficient and 95% confidence interval)

The Cox PH model with the aforementioned true relevant predictors showed that the whole model (*P* = <0.001), homozygous APOE-ɛ4 vs none (*P* = <0.001), MMSE score (*P* = <0.001), and serotonin (*P* = 0.02) violated the PH assumption. Consequently, we fitted the Cox PH model comprising the original predictors, the time-dependent MMSE score and serotonin, and stratification of the baseline hazard function for homozygous APOE-ɛ4 vs none. The result showed that over time, a one-SD increase in 5OH-IAA is significantly associated with a constant 42% decrease ACD risk (adjusted hazard ratio (HR) 0.58; 95% confidence interval (CI): 0.36 to 0.94, *P* = 0.025) (Fig. 2A). In line with the Cox PH model, the AFT model with the true relevant predictors showed that for each one-SD increase in 5OH-IAA, the logarithm of ACD survival time significantly increases by 0.21 (adjusted coefficient 0.21; 95% CI 0.02 to 0.40, *P* = 0.03) years (Fig. 2B). Other significant predictors of time-to-ACD were age, sex, homozygous APOE-ɛ4 vs none, heterozygous APOE-ɛ4 vs. none, MMSE score, physical active vs. inactive, TBI, and serotonin.

#### Bias Analysis

The *E*-values for 5OH-IAA were 2.27 and 1.27 for its adjusted HR of 0.58 (risk ratio, RR of 0.69) and upper CI of 0.94 (RR of 0.95), respectively. The observed adjusted HR of 0.58 could be explained away by an unmeasured confounder that is associated with both 5OH-IAA and time-to-ACD risk by a RR of 2.27-fold each, beyond the measured confounders, but weaker confounding could not do so. Furthermore, the upper CI could be moved to include one by an unmeasured confounder that was associated with both 5OH-IAA and time-to-ACD risk by a RR of 1.27-fold each, above and beyond the measured confounders, but weaker confounding could not do so. In addition, the association between 5OH-IAA and time-to-ACD risk was robust to the exclusion of cases within the first 1 year of follow-up, making it unlikely that the result is explained by reverse causality (Table A.4).

#### Alzheimer’s Disease Dementia

**Fig. 3:**
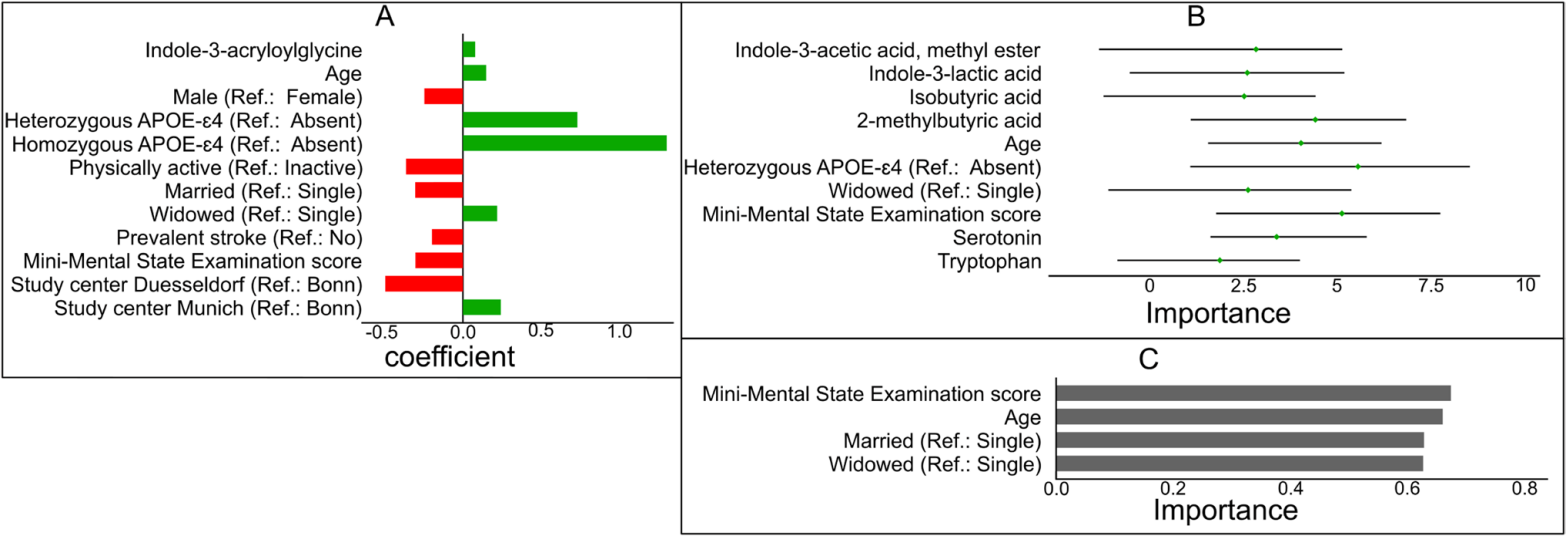
The true relevant predictors of Alzheimer’s disease dementia risk obtained from adaptive Least Absolute Shrinkage and Selection Operator logistic regression (green: positively associated predictors, red: negatively associated predictors) (**A**), random forest–based Boruta algorithm (Importance = median, minimum, and maximum *z*-scores of importance computed over multiple iterations) (**B**), and recursive feature elimination implemented using Naïve Bayes algorithm (Importance = area under the ROC curve (AUC) score importance) (**C**)

The 12 (six positive and six negative) non-zero predictors from aLASSO logistic regression were IAG, age, sex, MMSE score, homozygous APOE-ɛ4 vs none, heterozygous APOE-ɛ4 vs. none, married vs single, widowed vs. single, prevalent stroke vs. not, physical active vs. inactive, Düsseldorf vs. Bonn, and Munich vs. Bonn (Fig. 3A). The RF-Boruta confirmed 10 important predictors, ILA, IAA ME, isobutyric acid, 2-methylbutyric acid, age, MMSE score, homozygous APOE-ɛ4 vs none, widowed vs. single, serotonin, and tryptophan, as discriminating AD cases from non-cases (Fig. 3B). The RFE-NB showed that a four-predictor model with age, MMSE score, married vs single, widowed vs. single optimally discriminates AD cases from non-cases (Fig. 3C). Overall, 18 true relevant predictors discriminated AD cases from non-cases. This included five gut microbiome molecules (IAG, ILA, IAA ME, isobutyric acid, 2-methylbutyric acid) and 13 covariates (age, sex, MMSE score, homozygous APOE-ɛ4 vs none, heterozygous APOE-ɛ4 vs. none, married vs single, widowed vs. single, serotonin, tryptophan, prevalent stroke vs. not, physical active vs. inactive, study center Düsseldorf vs. Bonn, and study center Munich vs. Bonn). However, none of the four inflammatory markers discriminated AD cases from non-cases.

**Fig. 4:**
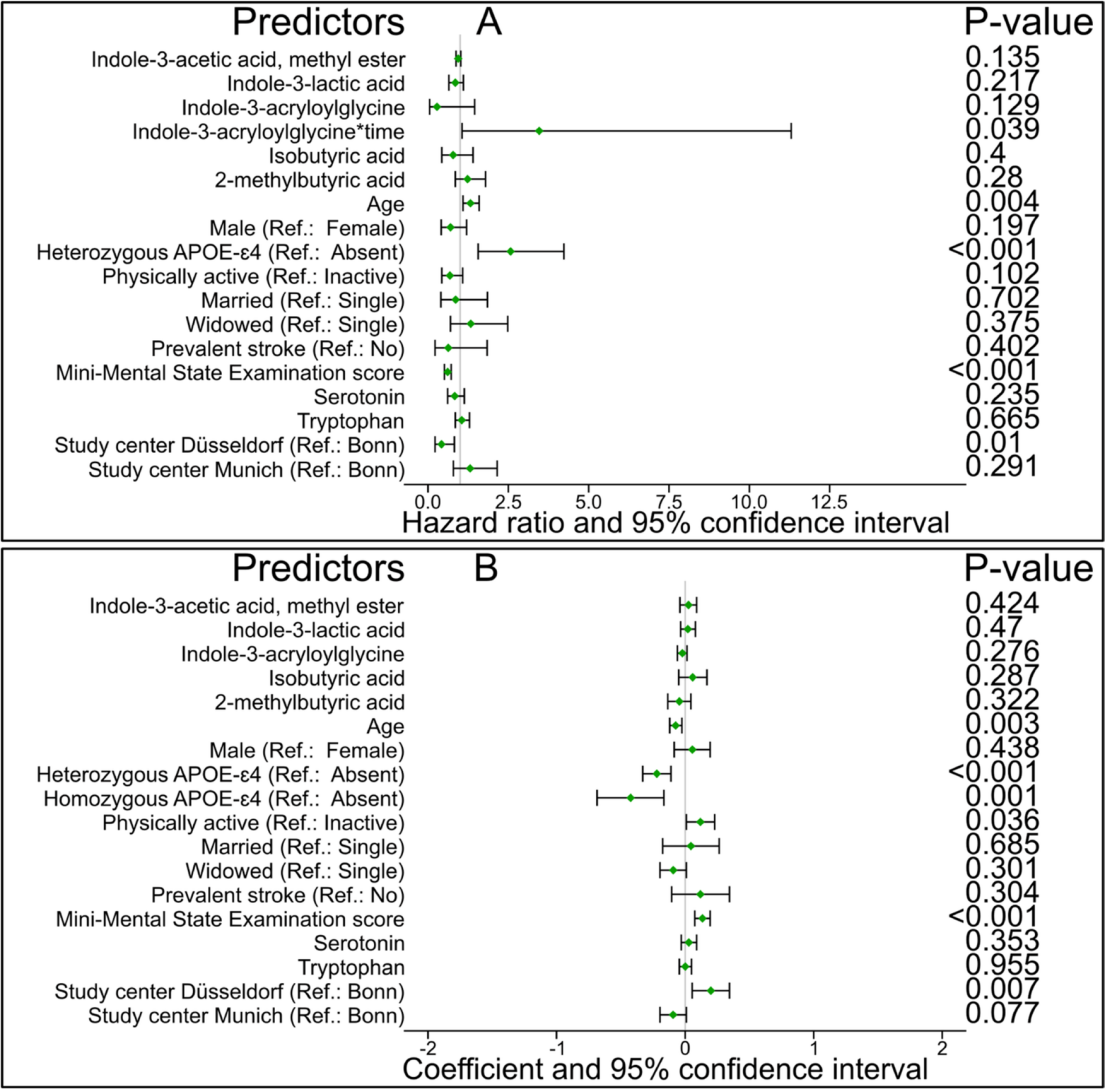
Association between true relevant predictors and time-to-Alzheimer’s disease dementia risk. **(A**) Weighted Cox proportional hazard model (hazard ratio and 95% confidence interval). **(B)** Weighted least-squares accelerated failure time (coefficient and 95% confidence interval)

The Cox PH model with the aforementioned true relevant predictors showed that the whole model (global test, *P* = <0.001), IAG (*P* = <0.001), and homozygous APOE-ɛ4 vs none (*P* = <0.001) violated the PH assumption. Consequently, we fitted the Cox PH model comprising the original predictors, time-dependent IAG, and stratification of the baseline hazard function for homozygous APOE-ɛ4 vs none. We observed that none of the primary predictors was associated with time-to-AD in the Cox PH (Fig. 4A) and AFT models (Fig. 4B). Consequently, no bias analysis was performed for AD. The significant predictors of time-to-AD were age, homozygous APOE-ɛ4 vs none, heterozygous APOE-ɛ4 vs. none, MMSE score, physical active vs. inactive, and study center Düsseldorf vs. Bonn.

## Discussion

The present epidemiological study leveraging a unique multicenter German cohort explored the relationship between the plasma levels of 19 gut microbiome molecules and four inflammatory markers and the risk of incident dementia. There were three observations. Firstly, seven (three ACD-related: 5OH-IAA, IBA, and 2-methylbutyric acid and five AD-related: IAG, ILA, IAA ME, isobutyric acid, and 2-methylbutyric acid) gut microbiome molecules discriminated incident dementia cases from non-cases. Secondly, decreased 5OH-IAA level was associated with elevated time-to-ACD. Thirdly, there was no association between inflammatory markers and the risk of either ACD or AD.

The relationship between IBA, IAG, ILA, and IAA ME and either ACD or AD risk has not been reported previously. Hence, the current study adds to the body of literature. Our observed relationship between 2-methylbutyric acid and AD risk is in consort with Wu et al. where fecal 2-methylbutyric acid was associated with AD risk in 55 individuals [22]. 2-Methylbutyric acid is a branched SCFA produced by microbial fermentation of isoleucine [50]. Another AD-related branched SCFA, isobutyric acid is produced by microbial fermentation of valine [50]. Isobutyric acid was previously associated with ACD [16]. *Bacteroides*, *Propionibacterium*, *Bacillus*, *Lactobacillus*, *Clostridium*, and *Escherichia coli* produce isobutyric acid and 2-methylbutyric acid [50]. The potential explanation for their relationship with dementia is that SCFAs modulate microglial activation, although the exact signaling pathways are not fully understood [51]. SCFAs also stimulate the expression of aryl hydrocarbon receptor (AhR) factors [52].

The first indole-containing tryptophan metabolite, ILA is secreted by *Bifidobacterium infantis* [53] and *Lactilactobacillus s*pecies [54], functioning as a potent activator of human AhR signaling [52]. The second, IBA is a precursor of IAA [55] produced by *Bifidobacterium*, *Lactilactobacillus*, *Clostridium*, and *Bacteroides* [54]. Furthermore, IAA ME is a metabolite of IAA produced by *Pseudomonas amygdali* [56] and IAG is synthesized from microbial tryptophan metabolism and host glycine conjugation [57]. The last, 5OH-IAA is the most compelling finding, as it was associated with time-to-ACD independent of several covariates and robust against bias. 5OH-IAA was previously associated with AD [23, 24]. Possible explanation for 5OH-IAA is that it is an agonist for AhR signaling [52, 58–60] and prevents the formation of amyloid beta plaques [61]. Thus, its low circulating levels suggest a reduction in these dementia-preventing properties. 5OH-IAA is synthesized mainly in the kidney and liver as the final serotonin catabolite in a two-step reaction, involving monoamine oxidase and aldehyde dehydrogenase [62]. Besides, it functions in the indole pathway [63]. Some gut bacteria such as *Pseudomonas* [64, 65] also directly synthesize its serotonin precursor. It is therefore unsurprising that 5OH-IAA is highly associated with the gut microbiome [66] and an emerging gut microbiome molecule [58–60, 67]. Interestingly, alteration in this host–gut microbiome co-molecule is implicated in neuropsychiatric conditions such as depression [67], epilepsy [68], and schizophrenia [69]. This suggests that the comorbidity of these conditions with dementia [70] may be partly underlined by the gut microbiome and 5OH-IAA.

Surprisingly, the gut microbiome de novo synthesized LPS and a measure of its long-term exposure (LBP) was not related to either ACD or AD. This is in contrast to the association of plasma LPS in 36 individuals [18] and plasma LBP in 636 individuals [19] with AD as well as the independent association of plasma LPS with cognitive decline in 127 individuals [71]. Moreover, other previously reported ACD-related gut microbiome molecules were saliva AA and PA in 51 individuals [15], fecal isovaleric acid in 107 individuals [16], and plasma IS in 24 individuals [17]. While Zhang et al. [18] was independent of age, André et al. [19] and Figueira et al. [15] were independent of a few covariates, Saji et al. [16] was not significant after adjusting for covariates, and Teruya et al. [17] was unadjusted for covariates. In addition, PA [21, 22] and 5OH-IAA [23, 24] have been consistently associated with AD, independent of age and sex. Yilmaz et al. observed the association in the saliva of 21 individuals [21], Wu et al. in the feces of 55 individuals [22]. Whiley et al. in the urine of 556 individuals [23], and Baker et al. in the brain of 25 individuals [24]. The fact most of the aforementioned molecules were not related to either ACD or AD suggests that the discrepancy between our findings and others is likely attributable to underlying differences in the study populations and methodologies. Moreover, the absence of association of any molecule with time-to-AD is unlikely due to statistical power since time-to-event analysis has greater statistical power than binary outcome analysis [72].

Unexpectedly, inflammatory markers, CRP, IL-1β, IL-6, and TNF-α did not discriminate dementia cases from non-cases. The absence of their association with dementia risk is unlikely to be due to intercorrelation of the markers, which is low, or covariate adjustment, since none was associated with dementia in the unadjusted analysis. This finding is in consort with recent reports [19, 29–31]. Two studies like ours investigated both ACD and AD [27, 29]. While the meta-analysis showed that CRP and IL-6 are associated with ACD and CRP with AD [27], the more recent study [29] did not observe an association of CRP with either ACD or AD. Considering that most recent studies [19, 30, 31] did not observe an association with other inflammatory markers casts doubt on the independent association between the circulating levels of this set of inflammatory markers and dementia risk. Nevertheless, this does not preclude the potential role of other inflammatory markers since systemic inflammation is the primary cause of BBB damage and often precedes dementia pathologies [73].

It is noteworthy that we confirmed previously reported determinants of dementia such as age [74], sex [16, 74], APOE-ɛ4 [16, 74, 75], MMSE [16, 22], and physical inactivity [2, 75]. In fact, we observed that age, sex, APOE-ɛ4, and MMSE were independent predictors of time-to-dementia risk. Additionally, our objectively measured habitual diet proxies that were negligible predictors of dementia agree with recent inconclusive evidence linking diet to dementia risk [76–78]. Furthermore, there are indications that the association between diet and dementia risk is driven by cardiovascular risk factors [2]. Since we adjusted for cardiovascular risk factors, it is likely that residual confounding by diet would be trivially small, despite SCFA and indole-containing tryptophan metabolites being diet-dependent. Furthermore, APOE-ɛ4 is a strong determinant of dementia and interacts with other risk factors [74]. Adjusting for APOE-ɛ4 status as a three-group variable ensures that its influence and related factors are properly accounted for.

There are several strengths of this study. It is a multicenter study; as such, these findings are generalizable to the broader German population. It is also one of the largest studies linking this set of biomarkers to dementia risk. Our analyses are sufficiently powered to produce valid and reliable results. Standardization of all continuous predictors reduces the impact of variation in the molecules and inflammatory markers thereby facilitating their comparison. Furthermore, our multivariable analysis ensured that we accounted for most of the inter-relationships among the molecules and inflammatory markers, covariates, and dementia risk. Our reporting of the effect estimates of primary predictors and covariates will improve the statistical power estimation of future studies. Finally, the case–cohort design affords the use of the subcohort for other health conditions since it is not case-matched. While this study is hypothesis-driven and yielded scientifically interesting and biologically plausible findings, it is important to acknowledge its limitations. This is an observational study; thus, it does not prove causal relationship between these molecules and dementia risk. Indeed, the potential causal relationship of 5OH-IAA with ACD risk may be explored by linking genetically predicted levels of 5OH-IAA with ACD risk in Mendelian randomization analysis. Besides, our finding may be different in other biospecimen. However, this seems unlikely for 5OH-IAA since there is a good agreement of its plasma, serum, and urine levels [60]. Moreover, we reported the impact of reverse causality and the strength of unmeasured confounders that are likely to explain away our findings. Despite these bias analyses and extensive adjustment for covariates, our results could still be biased due to measurement error and the use of two categories for some covariates. Our limited sample size precludes an intermediate step of internally validating and assessing the potential clinical utility of the true relevant predictors in a holdout dataset. Our findings should be confirmed in other studies, particularly in larger well-characterized and harmonized multicenter studies with broader microbiome metabolomics and a larger set of inflammatory markers. Since the levels of some of these dementia-related molecules are time-dependent [33], the relationship between their repeatedly measured levels and dementia risk should be investigated. Indeed, the AD-related molecules are interesting candidates to thoroughly elucidate in future biomarker-based research.

## Conclusion

In a relatively large cohort of older Germans, we observed that circulating concentrations of seven gut microbiome molecules are related to dementia risk, of which 5OH-IAA is associated with long-term ACD risk. These molecules underpin gut microbiome-host interactions in dementia occurrence. The modulation of these molecules such as through direct supplemental intake and probiotic consumption of their synthesizing bacteria may be crucially relevant in dementia’s multifactorial risk prevention and intervention strategies.

## Supplementary information

ESM 1 (XLSX 87 kb)

## Data Availability

The datasets generated during and/or analyzed during the current study are available from the corresponding author on reasonable request and subsequent approval of the principal coordinators of the AgeCoDe study.

## Abbreviations

AD: Alzheimer’s disease dementia
ACD: all-cause dementia
HbA1c: hemoglobin A1c
LPS: lipopolysaccharide
SCFA: short-chain fatty acids
BBB: blood-brain barrier
AA: acetic acid
PA: propionic acid
BA: butyric acid
HA: hexanoic acid
IS: indoxyl sulfate
LBP: LPS-binding protein
5OH-IAA: 5-hydroxyindole-3-acetic acid
IAA: indole-3-acetic acid
IACR: indoleacrylic acid
CRP: C-reactive protein
IL: interleukin
TNF-α: tumor necrosis factor-alpha
AgeCoDe: German study on Ageing, Cognition, and Dementia in Primary Care Patients
BMI: body mass index
CV: coefficient of variation
IAA ME: indole-3-acetic acid methyl ester
IPA: indole-3-propionic acid
IBA: indole-3-butyric acid
ILA: indole-3-lactic acid
ICARB: indole-3-carboxaldehyde
IAG: indole-3-acryloylglycine
DSM: Diagnostic and Statistical Manual of Mental Disorders
SIDAM: Structured Interview for Diagnosis of Dementia of Alzheimer type, Multi-infarct Dementia and Dementia of other etiology according to DSM-IV and ICD-10
DAG: directed acyclic graph
MMSE: Mini-Mental State Examination
APOE: apolipoprotein E
TBI: traumatic brain injury
MCAR: missing completely at random
MAR: missing at random
Cox PH: cox proportional hazards
aLASSO: adaptive Least Absolute Shrinkage and Selection Operator
RF-Boruta: random forest–based Boruta algorithm
RFE-NB: recursive feature elimination implemented using Naïve Bayes algorithm
AFT: accelerated failure time
SD: standard deviation
HR: hazard ratio
CI: confidence interval
RR: risk ratio
